# Domestication effect of reduced brain size is reverted when mink become feral

**DOI:** 10.1098/rsos.230463

**Published:** 2023-07-05

**Authors:** Ann-Kathrin Pohle, Andrzej Zalewski, Marion Muturi, Christian Dullin, Lucie Farková, Lara Keicher, Dina K. N. Dechmann

**Affiliations:** ^1^ Department of Migration, Max Planck Institute of Animal Behavior, Am Obstberg 1, 78315 Radolfzell, Germany; ^2^ Department for the Ecology of Animal Societies, Max Planck Institute of Animal Behavior, Bücklestraße 5a, 78467 Konstanz, Germany; ^3^ University of Konstanz, Universitätsstraße 10, 78457 Konstanz, Germany; ^4^ Mammal Research Institute, Polish Academy of Sciences, 17-230 Białowieża, Poland; ^5^ Department for Diagnostic and Interventional Radiology, University Medical Center Goettingen, Robert-Koch-Straße 40, 37075 Goettingen, Germany; ^6^ Department Translational Molecular Imaging, Max Planck Institute for Multidisciplinary Sciences, Herman-Rein-Straße 3, 37075 Goettingen, Germany; ^7^ Department for Diagnostic and Interventional Radiology, University Hospital Heidelberg, Im Neuenheimer Feld 420, 69120 Heidelberg, Germany; ^8^ Department of Zoology, Charles University, Viničná 7, 128 00 Prague, Czech Republic

**Keywords:** domestication, feralization, brain size, American mink

## Abstract

A typical consequence of breeding animal species for domestication is a reduction in relative brain size. When domesticated animals escape from captivity and establish feral populations, the larger brain of the wild phenotype is usually not regained. In the American mink (*Neovison vison*), we found an exception to this rule. We confirmed the previously described reduction in relative braincase size and volume compared to their wild North American ancestors in mink bred for their fur in Poland, in a dataset of 292 skulls. We then also found a significant regrowth of these measures in well-established feral populations in Poland. Closely related, small mustelids are known for seasonal reversible changes in skull and brain size. It seems that these small mustelids are able to regain the brain size, which is adaptive for living in the wild, and flexibly respond to selection accordingly.

## 1. Introduction

If and how much brain size matters for the evolutionary success of a species remains hotly debated [1–8]. To better understand the role of brain size linked to cognition, survival and evolutionary success, it is thus important to follow the evolution and flexibility of this trait. It was long assumed that a larger brain is advantageous [1–3,9] but research in recent decades has shown that the size and organization of the brain are much more flexible than previously believed, with entire clades secondarily reducing brain size in the course of evolution [4–6]. This is thought to be the result of a trade-off between the high energetic cost of the brain and cognitive ability [4–6].

Interesting in this context is the widely observed phenomenon where during domestication, animals often decrease in relative brain size (henceforth referred to as ‘domestication effect’) [10–16]. This is particularly due to the reduction of the relative size of brain regions involved in sight, sound and smell [17], and some studies suggest a trade-off in expensive tissues as a driver for this [18]. When populations of domesticated animals become feral, the original wild-type brain size is not recovered, and relative brain size usually remains smaller [12,19,20]. This irreversibility matches Dollo's law, which states that a trait once lost during evolution is lost forever [21], although some exceptions have been found [22–26] with at least one being presumably connected to a high amount of genetic diversity [22]. However, as feral animals presumably live in a much more complex, challenging and unpredictable environment than their domesticated ancestors, regaining larger brains could be considered adaptive [8]. A partial exception is a population of feral pigs in Sardinia, which have regained larger brains and higher neuron densities in the olfactory brain, but not all previous functions [27]. Further, a study on dingoes in 2018 found that they had larger brains and higher encephalization than domestic dogs, contrary to earlier studies on this species [28].

Studying the effect of domestication and potential recovery of brain size in feral animals is challenging due to the difficulty of obtaining measures of brain size in at least three sets of animals (wild, farm and feral). However, braincase size is a good proxy for brain size in many animals including several species of mustelids [29–32].

The American mink (*Neovison vison*) is a mustelid with a documented domestication effect on brain size [16,33]. American mink have been commonly bred for their fur since the 1920s and, following the typical pattern for domesticated animals, they have undergone an increase in body size and a decrease in relative brain size of up to 20% [33–35]. After the species was brought from America to Europe in the 1920s, it soon spread throughout Europe and became feral after escaping from fur farms and by intentional releases into the wild [36,37]. The American mink has been feral in Europe since the first half of the 1930s [36] and in Poland by the end of the 1970s [38]. Poland was populated in a wave-like structure, with mink entering the country from the northeast and northwest from Belarus, Lithuania and Russia, and potentially from additional countries such as Germany [38,39]. The species then spread throughout Poland from north to south, most likely due to escapees within Poland itself [38]. Since then, more than 40 generations have reproduced under selection by the natural environment. Age, origin, and development of these populations are known [36,38,40] and skull collections from all three phenotypes (wild, farm and feral) are available. The American mink thus offers the rare opportunity to compare the relative brain size of three phenotypes, wild from North America and farm and feral from Europe, to gain a better understanding of the flexibility and the role that total brain size plays in feral animals.

We investigated brain size changes as represented by the skull by comparing skull dimensions and braincase volumes of wild, farm and feral mink. Based on the fact that closely related small mustelids have brains that seasonally change in size indicating high flexibility in brain size [31], we hypothesized that mink would also have particularly flexible brains and perhaps be able to reduce and later regrow them. We expected that (i) ‘farm’ mink should have smaller relative brains than ‘wild’ mink, as measured by skull size and braincase volume, and that (ii) ‘feral’ mink should at least partly recover their relative brain size. With this study, we make an important contribution to the understanding of the domestication effect on brain size and thus the flexibility of this trait.

## 2. Methods

### 2.1. Skull collections

Skulls of wild American mink were obtained from the collection of the Cornell University Museum of Vertebrates (note that not all measurements could be taken from each skull; a detailed table with the sample sizes for each measurement can be found in the electronic supplementary material, table S1). We measured skulls of farm and feral American mink in the collection of the Mammal Research Institute PAS, Białowieża, Poland. Skulls from different populations in Poland and the USA were pooled per country after confirming there was no significant variation within the Polish or US datasets.

We only analysed data from skulls of individuals sampled in November (*n*_feral_ = 203, *n*_wild_ = 35 and *n*_farm_ = 54) as this was the time of year when farm-bred animals were sacrificed. All skulls from farm animals were from subadult animals born in the same year as that is when they are culled for their fur. We measured animals from a single month to avoid confounding effects of potential seasonal brain size changes, found in some small mammals, including mustelids, where young but fully grown animals have the largest braincase [31,41]. The braincase then shrinks reaching a minimum size in winter and regrows in spring [41–43]. The dataset of farm mink consisted only of first-year individuals while feral and wild mink also included older individuals. Since in species with seasonal brain size changes first-year animals have the largest relative skulls, including only them in the category—farm—that we expect to be smallest, would only weaken the effect we test for.

### 2.2. External skull measurements

We took linear measurements with callipers (accuracy ± 0.01 mm, Mahr IP67 MarCal 16EW) following LaPoint [31] and Schmidt [44] (electronic supplementary material, figure S1a–c). Condylobasal length (CBL) was measured from the most posterior point of the occipital condyles to the anterior of the premaxilla and braincase width (BW) was measured posterior from the zygomatic arches. For braincase height (BH), we took measurements left and right of the crest, omitting the auditory bullae on the ventral side of the skull. We then used the average of these two measurements to calculate the height and minimize the effect of the sagittal crest which is usually larger in males and continues to grow throughout their life. We standardized our measurements by the CBL of the skull to obtain relative measures independent of body size variation. CBL served as a proxy for body size [31].

### 2.3. Estimation of braincase volume

For estimating braincase volume (Vol), we placed the skull inside a cup on a scale (accuracy ± 0.01 g, Kern EMB; electronic supplementary material, figure S1d) and filled it with 2 mm carbon steel beads (AISI 1015, www.kugel-rollen.de) to obtain the weight of the beads needed to fill up the cavity. To determine how many beads filled up each cavity and to determine its volume we first weighed an increasing number of beads to establish a linear regression line between the number of beads and their accumulated weight [31]. We then quantified the number of beads in each brain case by taking the weight of the inserted beads and determining the bead count of the regression line at that weight. To calculate braincase volume, the volume of a single bead was multiplied by the number of beads. Finally, this value was divided by CBL^3^ to obtain relative values. All linear measurements were taken by A.-K.P., all volume measurements were done by M.M. See electronic supplementary material for determination of measurement error (electronic supplementary material, table S2).

### 2.4. MicroCT scanning

For the visual representation of skull changes, we scanned two feral and two farm American mink skulls at the Mammal Research Institute PAS, Białowieża, Poland with a Bruker microCT 1276 operated with the following settings: tube voltage 82 kV, tube current 200 µA, filter 1 mm Al, rotation steps 0.4°, detector binning 2 × 2 resulting in a total scanning time of 11 min and a reconstructed voxel size of 20.5 µm × 20.5 µm × 20.5 µm. Four scans were needed to cover the entire skull. The scans were automatically stitched and reconstructed using NRecon v1.7.4.2, the proprietary software of the device. A second batch of two wild mink skulls was scanned at the Institute for Diagnostic and Interventional Radiology, Goettingen, using a QuantumFX in vivo microCT (Perkin Elmer) operated with the following settings: tube voltage 70 kV, tube current 200 µA, field-of-view 40 mm × 40 mm and total acquisition time per step 2 min. The data were reconstructed with a pixel size of 78 µm × 78 µm × 78 µm. A custom-made software was used to automatically stitch the two scans needed to cover the entire skull. Rendering and generation of surface models were performed with VGStudioMax 3.1 (VolumeGraphics). Three-dimensional alignment of the surface models and measurement of the jaw length for normalization were performed in Scry 6.1 (custom-made three-dimensional render and analysis software). Calculation of the local Hausdorff distance was performed in Meshlab 2022.02.

### 2.5. Statistical analysis

We calculated the percentage of size difference (electronic supplementary material, tables S3 and S4) between each category (wild, farm and feral) using the median of each measurement. The median instead of the mean value was used due to differences in sample size. Detailed information on available sample size for each category and sex can be found in the electronic supplementary material, table S1. As females are smaller than males in the American mink [45], we included sex as an explanatory variable into all analyses. We then built linear models (package ‘lme4’) [46] to explore differences in relative Vol, relative BH and relative BW between our categories. Each measurement was the outcome variable in a separate model. We used a full- versus null-model comparison to confirm the importance of our predictor ‘category’ for this analysis. The null model consisted only of our measured outcome variables (relative Vol, BH and BW) with sex as the only predictor variable. The full model included sex and category as predictor variables. We tested the significance of category as a predictor using an analysis of variance (ANOVA) on the full and the null model and by comparing their Akaike information criterion (AIC) values. Summary tables were created using package ‘sjPlot’ [47]. We performed pairwise comparisons between each category per sex for CBL, relative Vol, BH and BW using package ‘emmeans’ [48]. Other packages used included ‘tidyverse’ [49] and ‘cowplot’ [50]. All analyses were done using R v. 4.0.3 [51] and RStudio v. 2023.3.1.446 [52].

## 3. Results

### 3.1. Category as a predictor enhances model performance

We found significant differences between the full and null models (ANOVA_vol_ *F* = 105.98, *p* < 0.0001; ANOVA_BH_ *F* = 54.708, *p* < 0.0001; ANOVA_BW_ *F* = 66.306, *p* < 0.0001) and AIC values confirmed that the full models performed better at explaining size differences in this dataset (ΔAIC_vol_ = 156.6, ΔAIC_BH_ = 90.03, ΔAIC_BW_ = 106.654). Thus, it was confirmed that category is a crucial predictor for this analysis.

### 3.2. Relative braincase volume is smallest in farm mink

**Figure 1.**
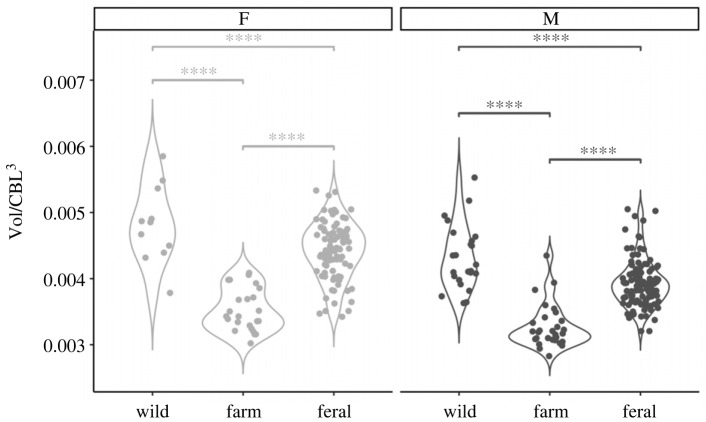
Violin plot comparing relative braincase volume (in mm^3^) in female (light grey) and male (dark grey) mink from wild (*n*_female_ = 11, *n*_male_ = 24), farm (*n*_female_ = 24, *n*_male_ = 29) and feral (*n*_female_ = 91, *n*_male_ = 110) populations. In both sexes, relative Vol was lowest in the farm population. Hash symbols indicate the level of significance between two compared categories within each sex (*p* ≤ 0.0001 = ***).

We found significant differences between all categories (table 1, figure 1; electronic supplementary material, table S5). As farm mink are bred to be large, their skulls were significantly larger in CBL (males: CBL_farm_ = 73.9 mm; females: CBL_farm_ = 66.8 mm) and absolute Vol (males: Vol_farm_ = 1275.4 mm^3^; females: Vol_farm_ = 1057.9 mm^3^) than those of wild (males: CBL_wild_ = 64.5 mm, Vol_wild_ = 1115.4 mm^3^; females: CBL_wild_ = 57.2 mm, Vol_wild_ = 901.3 mm^3^) and feral mink (males: CBL_feral_ = 68.8 mm, Vol_feral_ = 1272.3 mm^3^; females: CBL_feral_ = 61.1 mm, Vol_feral_ = 1020.9 mm^3^). However, once standardized by CBL, relative external and internal skull measures (Vol/CBL^3^, BH/CBL, BW/CBL) of wild and feral individuals were larger than those of farm individuals (electronic supplementary material, figure S9).

**Table 1.** Summary table of the linear model for relative braincase volume (Vol/CBL^3^) with sex and category as fixed effects. Intercept includes ‘wild’ and ‘females’. Number of observations = 289 (wild_males_ = 24, wild_females_ = 11, farm_males_ = 29, farm_females_ = 24, feral_males_ = 110, feral_females_ = 91). Estimates and confidence intervals displayed in this table were multiplied by 1000 to facilitate readability.

predictors	estimates	CI	*p*-value
(intercept)	4.78	4.64–4.93	<0.001
sex [M]	−0.45	−0.55 to −0.36	<0.001
category [farm]	−1.15	−1.32 to −0.98	<0.001
category [feral]	−0.39	−0.53 to −0.24	<0.001
*R*^2^/*R*^2^ adjusted	0.505/0.500		

**Figure 2.**
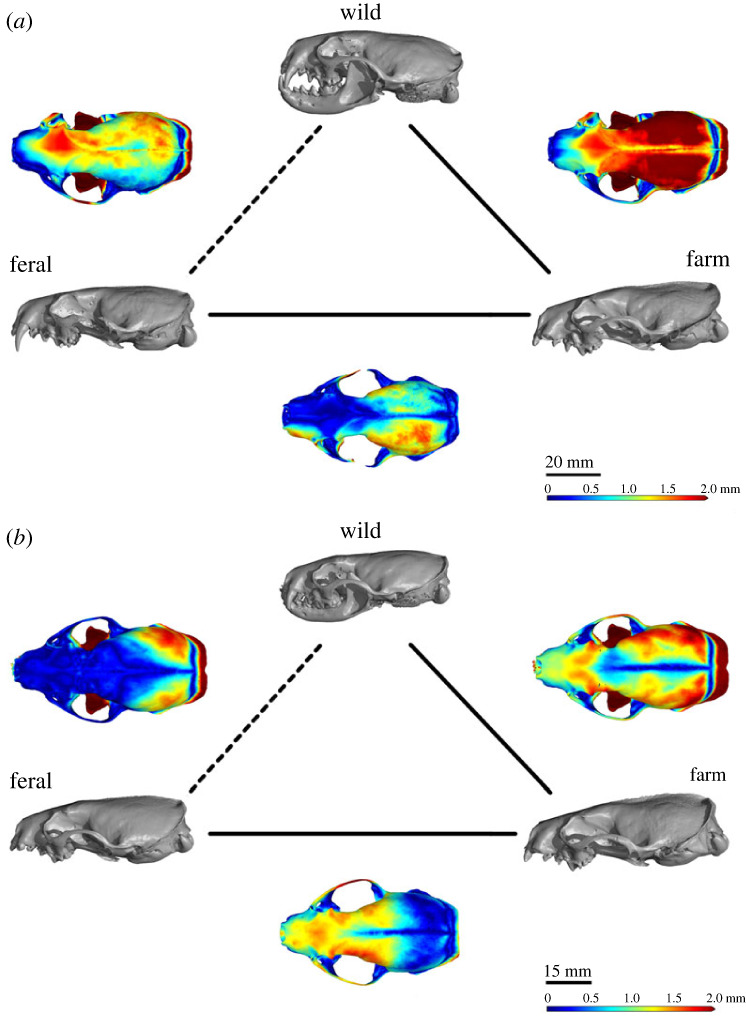
Visual representation of skull changes (representative skulls) in (*a*) males and (*b*) females. Skull microCT scans are shown in grey and labelled with their respective category (clockwise starting at the top: wild, farm and feral). Along the lines, we placed overlaps of those scans to illustrate in what areas the skulls differ. Blue colours indicate small differences and red colours indicate large differences. One representative skull per category and sex was chosen for visualization.

Our linear model explained 50% of the variance in relative Vol (adjusted *R*^2^-value = 0.50; table 1). It also confirmed a significant influence of sex and category on relative Vol. We found significant differences between all categories in both sexes (figure 1, table 1; electronic supplementary material, figure S3), with farm mink reducing relative Vol by 23.4% in males and 29.4% in females compared to their wild counterparts from America. Between farm and feral mink, we observed an increase in relative Vol (21.8% in males and 29.9% in females). Differences between wild and feral mink were least pronounced with relative Vol of feral animals 6.7% (males) and 8.2% (females) smaller than in wild individuals (detailed description in the electronic supplementary material, tables S3 and S4). See electronic supplementary material, table S5, for individual *p*-values of pairwise comparisons between each category and sex. Exact sample sizes for each sex and category can be found in the electronic supplementary material, table S1. A visual representation of differences in the braincase between each category can be viewed in figure 2.

### 3.3. Changes in relative braincase height and width are less pronounced

Our linear model explained 33% (adjusted *R*^2^-value = 0.33; electronic supplementary material, table S6) of the variance in relative BH (electronic supplementary material, table S6). In relative BH, we found significant differences between all categories similar to those found in relative Vol, though differences were at times less pronounced (electronic supplementary material, figures S4 and S5). Farm mink were between 7.5% and 7.9% smaller than their wild predecessors (males and females, respectively). After becoming feral, males increased by 3.4% in size and females by 8.9% (electronic supplementary material, tables S3 and S4). The difference between wild and feral females in relative BH was still significant (*p* = 0.0012; electronic supplementary material, table S7) but feral females differed by less than 1% from the ‘original’ BH of wild females. Feral males were about 4.37% smaller in relative BH than wild males (electronic supplementary material, tables S3 and S4, for changes in percentage; electronic supplementary material, tables S6 and S7, for details of model summary and pairwise comparisons).

Comparisons of relative BW between categories were less pronounced than those in relative Vol and BH. The difference between feral and wild males and females in relative BW was still significant, though less pronounced than between other categories. Detailed results of our linear model regarding BW are presented in the electronic supplementary material, figures S6 and S7, and tables S8 and S9.

## 4. Discussion

Our investigation supported previous evidence about the domestication effect on skull and thus brain size of wild and farm mink and provided new insights about the irreversibility of this process. We confirmed a decrease in relative braincase volume of up to 29% during domestication, but we also found that feral animals regained 22–30% in relative braincase volume compared to farm animals. This recovery of relative braincase size in feral animals shows that species differ in the short-term flexibility of brain size.

To understand how these patterns emerged, we compared absolute with relative values: CBL, as a proxy for body size, increased significantly from wild to farm animals (electronic supplementary material, figure S8), most likely due to breeding for size in the fur industry [53,54]. Absolute braincase size, however, only partially increased (electronic supplementary material, figure S8), resulting in a significantly smaller braincase relative to CBL (electronic supplementary material, figure S9). After mink became feral, CBL (and body size) decreased, but absolute braincase size remained roughly the same. This resulted in a larger braincase relative to CBL. A change in relative brain size is usually the indirect result of faster or slower changes in body size compared to the brain [6]. Contrary to previous literature [10–15] this suggests that relative brain size can in fact recover in feral animals.

The decrease of 23–29% (electronic supplementary material, tables S3 and S4) we found in braincase size fitted the general magnitude of the domestication effect on brain size [12,14,33], and differed only slightly from the 20% reported by Kruska in 1996, based on brain mass [33], even though other studies have found less or no decrease in braincase volume in this species [35,55]. Previous studies indicate that the reduction of brain size in the course of domestication is the result due to strong reductions of entire brain regions [15,17,33,56] which then also explains why brain size is usually not recovered in feral populations. In order to better understand why the American mink differs in this pattern, it will be necessary to study brains of wild, feral and farmed animals. In the case of the feral Sardinian pigs, certain brain regions did recover, but without the expression of important functional proteins connected to the immaculate sense of smell in wild boars [27]. Small mustelids that are closely related to the American mink have been found to exhibit Dehnel's Phenomenon, reversible skull and thus brain size changes between seasons, indicating that mustelid brains may be particularly flexible [30]. American farm mink have smaller brain regions relevant for motor functions but no loss of brain regions [33,56]. Combining MRI brain scans of the American mink with behavioural studies on problem-solving skills will help to detect possible changes in brain structures and associated consequences.

Changes in brain size pose yet another trade-off and are associated with disadvantages as well as benefits. Brain tissue is expensive to maintain and thus brain reduction can be beneficial to reduce energetic costs [5], indicating that larger brains are not always better. Brains of domesticated animals—though smaller—may merely be adapted to a different niche, which is their captive environment. Domesticated animals such as rats and dogs have higher social-cognitive, learning and memory capabilities than their wild counterparts [57–59]. However, brain size has also predicted how successful mammals were when introduced to novel environments, with higher rates of success in larger brained animals [3]. Further, increased brain size is connected to increased urban tolerance in birds [9] and a recent study even showed that larger brained animals were more likely to survive an extinction wave in the Late Quaternary (approximately 115 ka–500 years ago) [7]. For an invasive species such as the American mink, quickly adapting aspects of the body and brain to a new environment may provide major advantages. In guppies, laboratory-reared individuals also have smaller relative brains [60] yet larger relative brain size in this species is linked to improved behavioural predator response [61]. Larger brain volume is linked to technical problem-solving in a study including 39 mammalian carnivore species [62]. Comparative studies of the energy expenditure of wild, farm and feral American mink could help understand if a relatively larger brain is energetically more costly, whether there are cognitive or behavioural trade-offs, and if and how they adjust their behaviour accordingly. In this context, investigating the expensive tissue hypothesis, which was suggested to play a role in the domestication effect on the brain and gut size of cats [18], could provide further insight.

This study provides new important insight for future domestication research. As the domestication effect on the relative brain size has often been found to be irreversible [12,20] with only a few exceptions [27,28], this study reveals another possible exception to the rule. Based on these results, it seems possible to find similar patterns in other species. If so, analysis on the similarities and differences between species and exploring of other hypotheses such as the expensive tissue hypothesis [18] might provide more answers to why and how such reversibility can arise and why Dollo's law [21] does not always apply in the context of domestication. It may be that species with flexible brain sizes such as small mustelids, shrews and moles are able to produce significant changes in size without loosing capabilities connected to these brain regions, so that in this case even a reversal to the wild condition in feral forms is more likely [27,31,63,64].

In conclusion, we found evidence for an almost complete reversal of the domestication effect on relative braincase size in feral American mink. Domestication has driven morphological changes in several traits, such as fur quality or body size [53,54], but one of the most strongly affected is the brain. After almost a century of domestication, relative brain case volume decreased while the absolute length of the skull increased. After escapees established feral populations in Poland by the late 1970s [38], relative braincase volume nearly recovered after less than 50 years in the wild.

Throughout long periods of adaptation, evolution has shaped brain size and structure in multiple ways, creating organisms specialized to their unique environment. The idea that bigger brains are not always better has already been shown in the case of secondary brain reduction in bats [5]—even though larger brains are still often viewed as providing evolutionary advantage [1,3]. Whether or not brain size determines the success of a species—and if so, how—remains debatable. Here, we provide evidence that brains are subject to selective pressures and just as—or even more—flexible as any other organ. This flexibility of relative brain size opens new opportunities to further investigate what the ‘optimal’ brain size for certain species is in certain contexts, how brain size evolves, and why it differs between species.

## Data Availability

The data generated for this study are publicly available on Edmond from MPG: https://doi.org/10.17617/3.SFXRZN [65]. The relevant code for this research work is stored in GitHub: https://github.com/ann-kathrinpohle/reverted-domestication-effect-american-mink.git and has been archived within the Zenodo repository: https://doi.org/10.5281/zenodo.8029739 [66]. Electronic supplementary material is available on Figshare [67].

## References

[1] Anderson B. 1993 Evidence from the rat for a general factor that underlies cognitive performance and that relates to brain size: intelligence? Neurosci. Lett. **153**, 98-102. (10.1016/0304-3940(93)90086-Z) 8510832

[2] Jerison HJ. 1973 Evolution of the brain and intelligence. New York, NY: Academic Press.

[3] Sol D, Bacher S, Reader SM, Lefebvre L. 2008 Brain size predicts the success of mammal species introduced into novel environments. Am. Nat. **172**, S63-S71. (10.1086/588304) 18554145

[4] Köhler M, Moyà-Solà S. 2004 Reduction of brain and sense organs in the fossil insular bovid *Myotragus*. Brain Behav. Evol. **63**, 125-140. (10.1159/000076239) 14726622

[5] Safi K, Seid MA, Dechmann DKN. 2005 Bigger is not always better: when brains get smaller. Biol. Lett. **1**, 283-286. (10.1098/rsbl.2005.0333) 17148188 PMC1617168

[6] Smaers JB, Dechmann DKN, Anjali G, Christophe S, Safi K. 2012 Comparative analyses of evolutionary rates reveal different pathways to encephalization in bats, carnivorans, and primates. Proc. Natl Acad. Sci. USA **109**, 18 006-18 011. (10.1073/pnas.1212181109) 23071335 PMC3497830

[7] Dembitzer J, Castiglione S, Raia P, Meiri S. 2022 Small brains predisposed Late Quaternary mammals to extinction. Sci. Rep. **12**, 3453. (10.1038/s41598-022-07327-9) 35361771 PMC8971383

[8] Marino L. 2005 Big brains do matter in new environments. Proc. Natl Acad. Sci. USA **102**, 5306-5307. (10.1073/pnas.0501695102) 15811939 PMC556226

[9] Sayol F, Sol D, Pigot AL. 2020 Brain size and life history interact to predict urban tolerance in birds. Front. Ecol. Evol. **8**, 58. (10.3389/fevo.2020.00058)

[10] Castiglione S, Serio C, Piccolo M, Mondanaro A, Melchionna M, Di Febbraro M, Sansalone G, Wroe S, Raia P. 2021 The influence of domestication, insularity and sociality on the tempo and mode of brain size evolution in mammals. Biol. J. Linn. Soc. **132**, 221-231. (10.1093/biolinnean/blaa186)

[11] Guay PJ, Iwaniuk AN. 2008 Captive breeding reduces brain volume in waterfowl (Anseriformes). Condor **110**, 276-284. (10.1525/cond.2008.8424)

[12] Kruska D. 2005 On the evolutionary significance of encephalization in some eutherian mammals: effects of adaptive radiation, domestication, and feralization. Brain Behav. Evol. **65**, 73-108. (10.1159/000082979) 15627722

[13] Wilkins AS, Wrangham RW, Fitch WT. 2014 The ‘Domestication Syndrome’ in mammals: a unified explanation based on neural crest cell behavior and genetics. Genetics **197**, 795-808. (10.1534/genetics.114.165423) 25024034 PMC4096361

[14] Zeder MA. 2015 Core questions in domestication research. Proc. Natl Acad. Sci. USA **112**, 3191-3198. (10.1073/pnas.1501711112) 25713127 PMC4371924

[15] Zeder M. 2012 The domestication of animals. Rev. Anthropol. **9**, 321-327. (10.1080/00988157.1982.9977605)

[16] Balcarcel AM, Geiger M, Clauss M, Sánchez-Villagra MR. 2022 The mammalian brain under domestication: discovering patterns after a century of old and new analyses. J. Exp. Zool. Part B Mol. Dev. Evol. **338**, 460-483. (10.1002/jez.b.23105) PMC9787656 34813150

[17] Callaway E. 2016 When chickens go wild. Nature **529**, 270-273. (10.1038/529270a) 26791702

[18] Lesch R, Kotrschal K, Kitchener AC, Fitch WT, Kotrschal A. 2022 The expensive-tissue hypothesis may help explain brain-size reduction during domestication. Commun. Integr. Biol. **15**, 190-192. (10.1080/19420889.2022.2101196) 35957842 PMC9359384

[19] Kruska D, Röhrs M. 1974 Comparative-quantitative investigations on brains of feral pigs from the Galapagos Islands and of European domestic pigs. Z. Anat. Entwicklungsgesch. **144**, 61-73. (10.1007/BF00518633) 4851103

[20] Niego A, Benítez-Burraco A. 2021 Are feralization and domestication truly mirror processes? Ethol. Ecol. Evol. **34**, 557-590. (10.1080/03949370.2021.1975314)

[21] Gould SJ. 1970 Dollo on Dollo's law: irreversibility and the status of evolutionary laws. J. Hist. Biol. **3**, 189-212. (10.1007/BF00137351) 11609651

[22] Visser B et al*.* 2021 Phenotypic plasticity explains apparent reverse evolution of fat synthesis in parasitic wasps. Sci. Rep. **11**, 7751. (10.1038/s41598-021-86736-8) 33833245 PMC8032832

[23] Lynch VJ, Wagner GP. 2010 Did egg-laying boas break Dollo's law? Phylogenetic evidence for reversal to oviparity in sand boas (*Eryx*: Boidae). Evolution **64**, 207-216. (10.1111/j.1558-5646.2009.00790.x) 19659599

[24] Wiens JJ. 2011 Re-evolution of lost mandibular teeth in frogs after more than 200 million years, and re-evaluating Dollo's law. Evolution **65**, 1283-1296. (10.1111/j.1558-5646.2011.01221.x) 21521189

[25] Kohlsdorf T, Wagner GP. 2006 Evidence for the reversibility of digit loss: a phylogenetic study of limb evolution in *Bachia* (Gymnophthalmidae: Squamata). Evolution **60**, 1896-1912. (10.1111/j.0014-3820.2006.tb00533.x) 17089974

[26] Marshall CR, Raff EC, Raff RA. 1994 Dollo's law and the death and resurrection of genes. Proc. Natl Acad. Sci. USA **91**, 12 283-12 287. (10.1073/pnas.91.25.12283) PMC45421 7991619

[27] Maselli V, Polese G, Larson G, Raia P, Forte N, Rippa D, Ligrone R, Vicidomini R, Fulgione D. 2013 A dysfunctional sense of smell: the irreversibility of olfactory evolution in free-living pig. Evol. Biol. **41**, 229-239. (10.1007/s11692-013-9262-3)

[28] Smith B, Lucas T, Norris R, Henneberg M. 2018 Brain size/body weight in the dingo (*Canis dingo*): comparisons with domestic and wild canids. Aust. J. Zool. **65** 292-301. (10.1071/ZO17040)

[29] Isler K, Kirk EC, Miller JMA, Albrecht GA, Gelvin BR, Martin RD. 2008 Endocranial volumes of primate species: scaling analyses using a comprehensive and reliable data set. J. Hum. Evol. **55**, 967-978. (10.1016/j.jhevol.2008.08.004) 18817943

[30] Iwaniuk AN, Nelson JE. 2002 Can endocranial volume be used as an estimate of brain size in birds? Can. J. Zool. **80**, 16-23. (10.1139/z01-204)

[31] LaPoint S, Keicher L, Wikelski M, Zub K, Dechmann DKN. 2017 Growth overshoot and seasonal size changes in the skulls of two weasel species. R. Soc. Open Sci. **4**, 160947. (10.1098/rsos.160947) 28280592 PMC5319358

[32] Lázaro J, Hertel M, Sherwood CC, Muturi M, Dechmann DKN. 2018 Profound seasonal changes in brain size and architecture in the common shrew. Brain Struct. Funct. **223**, 2823-2840. (10.1007/s00429-018-1666-5) 29663134 PMC5995987

[33] Kruska D. 1996 The effect of domestication on brain size and composition in the mink (*Mustela vison*). J. Zool. **239**, 645-661. (10.1111/j.1469-7998.1996.tb05468.x)

[34] Pagh S et al. 2019 Methods for the identification of farm escapees in feral mink (*Neovison vison*) populations. PLoS ONE **14**, e0224559. (10.1371/journal.pone.0224559) 31710608 PMC6852605

[35] Kruska D, Sidorovich VE. 2003 Comparative allometric skull morphometrics in mink (*Mustela vison* Schreber, 1777) of Canadian and Belarus origin; taxonomic status. Mamm. Biol. **68**, 257-276. (10.1078/1616-5047-00095)

[36] Bonesi L, Palazón S. 2007 The American mink in Europe: status, impacts, and control. Biol. Conserv. **134**, 470-483. (10.1016/j.biocon.2006.09.006)

[37] Fraser EJ, Lambin X, Travis JMJ, Harrington LA, Palmer SCF, Bocedi G, Macdonald DW. 2015 Range expansion of an invasive species through a heterogeneous landscape—the case of American mink in Scotland. Divers. Distrib. **21**, 888-900. (10.1111/ddi.12303)

[38] Brzeziński M, Żmihorski M, Zarzycka A, Zalewski A. 2019 Expansion and population dynamics of a non-native invasive species: the 40-year history of American mink colonisation of Poland. Biol. Invasions **21**, 531-545. (10.1007/s10530-018-1844-7)

[39] Brzeziński M, Marzec M. 2003 The origin, dispersal and distribution of the American mink *Mustela vison* in Poland. Acta Theriol. **48**, 505-514. (10.1007/BF03192496)

[40] Zalewski A, Michalska-Parda A, Bartoszewicz M, Kozakiewicz M, Brzeziński M. 2010 Multiple introductions determine the genetic structure of an invasive species population: American mink *Neovison vison* in Poland. Biol. Conserv. **143**, 1355-1363. (10.1016/j.biocon.2010.03.009)

[41] Dechmann DKN, LaPoint S, Dullin C, Hertel M, Taylor JRE, Zub K, Wikelski M. 2017 Profound seasonal shrinking and regrowth of the ossified braincase in phylogenetically distant mammals with similar life histories. Sci. Rep. **7**, 42443. (10.1038/srep42443) 28211896 PMC5304206

[42] Dehnel A. 1949 Studies on the genus *Sorex* L. Ann. Univers. Marie Curie-Skłodowska Section C **5**, 17-102.

[43] Pucek Z. 1965 Seasonal and age changes in the weight of internal organs of shrews. Acta Theriol. **10**, 369-438. (10.4098/AT.arch.65-31)

[44] Schmidt K. 1992 Skull variability of *Mustela nivalis* Linnaeus, 1766 in Poland. Acta Theriol. **37**, 141-162. (10.4098/AT.arch.92-15)

[45] Zalewski A, Bartoszewicz M. 2012 Phenotypic variation of an alien species in a new environment: the body size and diet of American mink over time and at local and continental scales. Biol. J. Linn. Soc. **105**, 681-693. (10.1111/j.1095-8312.2011.01811.x)

[46] Bates D, Mächler M, Bolker B, Walker S. 2015 Fitting linear mixed-effects models using lme4. J. Stat. Softw. **67**, 1-48. (10.18637/jss.v067.i01)

[47] Lüdecke D. 2023 sjPlot: data visualization for statistics in social science. R package version 2.8.14.

[48] Lenth RV. 2021 emmeans: estimated marginal means, aka least-squares means. R package version 1.7.1-1.

[49] Wickham H et al. 2019 Welcome to the tidyverse. J. Open Source Softw. **4**, 1686. (10.21105/joss.01686)

[50] Wilke C. 2020 cowplot: streamlined plot theme and plot annotations for ‘ggplot2’. R package version 1.1.1.

[51] R Core Team. 2022 R: a language and environment for statistical computing. Vienna, Austria: R Foundation for Statistical Computing.

[52] RStudio Team. 2023 RStudio: integrated development environment for R. Vienna, Austria: R Foundation for Statistical Computing.

[53] Lagerkvist G. 1997 Economic profit from increased litter size, body weight and pelt quality in mink (*Mustela vison*). Acta Agric. Scand. Sect. A **47**, 57-63. (10.1080/09064709709362370)

[54] Karimi K, Sargolzaei M, Plastow GS, Wang Z, Miar Y. 2018 Genetic and phenotypic parameters for litter size, survival rate, gestation length, and litter weight traits in American mink. J. Anim. Sci. **96**, 2596-2606. (10.1093/jas/sky178) 29726960 PMC6095447

[55] Tamlin AL, Bowman J, Hackett DF. 2009 Separating wild from domestic American mink *Neovison vison* based on skull morphometries. Wildlife Biol. **15**, 266-277. (10.2981/08-004)

[56] Kruska D. 1988 Mammalian domestication and its effect on brain structure and behavior. In Intelligence and evolutionary biology (eds HJ Jerison, I Jerison), pp. 211-250. Berlin, Germany: Springer.

[57] Boice R. 1970 Effect of domestication on avoidance learning in the Norway rat. Psychon. Sci. **18**, 13-14. (10.3758/BF03332302)

[58] Hare B, Brown M, Williamson C, Tomasello M. 2002 The domestication of social cognition in dogs. Science **298**, 1634-1636. (10.1126/science.1072702) 12446914

[59] Boice R. 1972 Some behavioral tests of domestication in Norway rats*.* Behaviour **42**, 198-230. (10.1163/156853972X00275)

[60] Burns JG, Saravanan A, Rodd FH. 2009 Rearing environment affects the brain size of guppies: lab-reared guppies have smaller brains than wild-caught guppies. Ethology **115**, 122-133. (10.1111/j.1439-0310.2008.01585.x)

[61] van der Bijl W, Thyselius M, Kotrschal A, Kolm N. 2015 Brain size affects the behavioural response to predators in female guppies (*Poecilia reticulata*). Proc. R. Soc. B **282**, 20151132. (10.1098/rspb.2015.1132) PMC4528528 26203003

[62] Benson-Amram S, Dantzer B, Stricker G, Swanson EM, Holekamp KE. 2016 Brain size predicts problem-solving ability in mammalian carnivores. Proc. Natl Acad. Sci. USA **113**, 2532-2537. (10.1073/pnas.1505913113) 26811470 PMC4780594

[63] Lazaro J, Hertel M, Muturi M, Dechmann D. 2019 Seasonal reversible size changes in the braincase and mass of common shrews are flexibly modified by environmental conditions. Sci. Rep. **9**, 2489. (10.1038/s41598-019-38884-1) 30792434 PMC6385354

[64] Nováková L, Lázaro J, Muturi M, Dullin C, Dechmann DKN. 2022 Winter conditions, not resource availability alone, may drive reversible seasonal skull size changes in moles*.* R. Soc. Open Sci. **9**, 220652. (10.1098/rsos.220652) 36133148 PMC9449468

[65] Pohle A-K, Zalewski A, Muturi M, Dullin C, Farková L, Keicher L, Dechmann DKN. 2023 Domestication effect of reduced brain size is reverted when mink become feral. Edmond. (10.17617/3.SFXRZN) PMC10320332 37416828

[66] Pohle A-K, Zalewski A, Muturi M, Dullin C, Farková L, Keicher L, Dechmann DKN. 2023 Code for: Domestication effect of reduced brain size is reverted when mink become feral. Zenodo. (10.5281/zenodo.8029739) PMC10320332 37416828

[67] Pohle A-K, Zalewski A, Muturi M, Dullin C, Farková L, Keicher L, Dechmann DKN. 2023 Domestication effect of reduced brain size is reverted when mink become feral. Figshare. (10.6084/m9.figshare.c.6708315) PMC10320332 37416828

